# Segmentation-guided multi-modal registration of liver images for dose estimation in SIRT

**DOI:** 10.1186/s40658-022-00432-8

**Published:** 2022-01-25

**Authors:** Xikai Tang, Esmaeel Jafargholi Rangraz, Richard’s Heeren, Walter Coudyzer, Geert Maleux, Kristof Baete, Chris Verslype, Mark J. Gooding, Christophe M. Deroose, Johan Nuyts

**Affiliations:** 1 Nuclear Medicine and Molecular Imaging, KU Leuven, Leuven, Belgium; 2 Radiology, KU Leuven, Leuven, Belgium; 3 Nuclear Medicine, University Hospitals Leuven, Leuven, Belgium; 4 Radiology, University Hospitals Leuven, Leuven, Belgium; 5 Digestive Oncology, University Hospitals Leuven, Leuven, Belgium; 6 Medical Imaging Research Center (MIRC), UZ Herestraat 49, Box 7003, 3000 Leuven, Belgium; 7 Mirada Medical Ltd, Oxford, UK; 8 Quirem Medical B.V., Deventer, The Netherlands

**Keywords:** Selective internal radiation therapy (SIRT), Liver registration, Convolutional neural network (CNN), Internal dosimetry, Multi-modality images

## Abstract

**Purpose:**

Selective internal radiation therapy (SIRT) requires a good liver registration of multi-modality images to obtain precise dose prediction and measurement. This study investigated the feasibility of liver registration of CT and MR images, guided by segmentation of the liver and its landmarks. The influence of the resulting lesion registration on dose estimation was evaluated.

**Methods:**

The liver segmentation was done with a convolutional neural network (CNN), and the landmarks were segmented manually. Our image-based registration software and its liver-segmentation-guided extension (CNN-guided) were tuned and evaluated with 49 CT and 26 MR images from 20 SIRT patients. Each liver registration was evaluated by the root mean square distance (RMSD) of mean surface distance between manually delineated liver contours and mass center distance between manually delineated landmarks (lesions, clips, etc.). The root mean square of RMSDs (RRMSD) was used to evaluate all liver registrations. The CNN-guided registration was further extended by incorporating landmark segmentations (CNN&LM-guided) to assess the value of additional landmark guidance. To evaluate the influence of segmentation-guided registration on dose estimation, mean dose and volume percentages receiving at least 70 Gy (V70) estimated on the ^99m^Tc-labeled macro-aggregated albumin (^99m^Tc-MAA) SPECT were computed, either based on lesions from the reference ^99m^Tc-MAA CT (reference lesions) or from the registered floating CT or MR images (registered lesions) using the CNN- or CNN&LM-guided algorithms.

**Results:**

The RRMSD decreased for the floating CTs and MRs by 1.0 mm (11%) and 3.4 mm (34%) using CNN guidance for the image-based registration and by 2.1 mm (26%) and 1.4 mm (21%) using landmark guidance for the CNN-guided registration. The quartiles for the relative mean dose difference (the V70 difference) between the reference and registered lesions and their correlations [25th, 75th; r] are as follows: [− 5.5% (− 1.3%), 5.6% (3.4%); 0.97 (0.95)] and [− 12.3% (− 2.1%), 14.8% (2.9%); 0.96 (0.97)] for the CNN&LM- and CNN-guided CT to CT registrations, [− 7.7% (− 6.6%), 7.0% (3.1%); 0.97 (0.90)] and [− 15.1% (− 11.3%), 2.4% (2.5%); 0.91 (0.78)] for the CNN&LM- and CNN-guided MR to CT registrations.

**Conclusion:**

Guidance by CNN liver segmentations and landmarks markedly improves the performance of the image-based registration. The small mean dose change between the reference and registered lesions demonstrates the feasibility of applying the CNN&LM- or CNN-guided registration to volume-level dose prediction. The CNN&LM- and CNN-guided registrations for CTs can be applied to voxel-level dose prediction according to their small V70 change for most lesions. The CNN-guided MR to CT registration still needs to incorporate landmark guidance for smaller change of voxel-level dose estimation.

## Introduction

Selective internal radiation therapy (SIRT) or radioembolization is increasingly applied for the treatment of surgically unresectable primary liver malignancies and secondary metastases. During this treatment, microspheres loaded with β-emitting radionuclides, including yttrium-90 (^90^Y) or holmium-166 (^166^Ho), are infused into selected branches of the hepatic artery according to the vascular anatomy mapped by angiography [1]. Since the selected branches dominate the blood supply to tumors [2], these radioactive microspheres are trapped within the tumors. The high energy, small tissue penetrating range, and concentration in tumors allow these microspheres to deposit higher energy per mass in tumors, while preventing healthy liver parenchyma dysfunction by limiting their irradiation..

In the SIRT planning, the absorbed dose is used to measure the amount of energy per mass (in Gy or J/kg) from ionizing radiation deposited in a volume of interest (VOI), including tumors and healthy liver parenchyma. It serves as a toxicity indicator for tumors and normal tissues and a criterion for determining the amount of injected activity. There are several different methods to determine the injected activity, including mono-compartment [3] and multi-compartment [4] methods and voxel-based approaches [3]. The dose calculation better reflects the underlying biology as the VOI changes from the volume level (the whole liver, tumors, and non-tumoral parts) to the voxel level.

During the pre- and post-treatment studies, multi-modality images are acquired for VOI delineation and dose calculation. In the pre-treatment study, a SPECT/CT scan is performed shortly after administration of ^99m^Tc-labeled macro-aggregated albumin (^99m^Tc-MAA) particles into selected branches of hepatic artery to mimic the activity distribution of ^90^Y microspheres in the liver [5]. Cone beam CT (CBCT) images are used for delineation of liver perfusion territories (LPTs) [6]. ^18^F-fluorodeoxyglucose ([^18^F]FDG) or [^68^Ga]DOTATATE PET/CT scans are performed for [^18^F]FDG- or [^68^Ga]DOTATATE-avid tumors. Contrast-enhanced and diffusion-weighted MR images are acquired for tumors that are not [^18^F]FDG- or [^68^Ga]DOTATATE-avid. Following the administration of ^90^Y-microspheres, a post-treatment study is performed to obtain the PET/CT or PET/MR images of the actual activity distribution inside the liver [7]. This PET image can be used to compute the absorbed dose in different VOIs for evaluation of the treatment irradiation distribution.

Registration of multi-modality images plays an essential role in information integration for SIRT dosimetry. Different methods of liver registration for intra-modality (CT or MR) and inter-modality images (CT and MR) have been studied. These methods include surface-based [8–11], vessel-based [9, 11, 12], intensity-based [10, 13, 14], and segmentation-based registrations [15, 16]. Some studies combine both liver surfaces and vascular structures for better registration of tumors inside the liver [9, 11], since most tumors are found near vessels. Most intensity-based methods adopt mutual information for image similarity measurement. The segmentation-based methods use liver segmentations obtained either with histogram-based thresholding [15] or with a convolutional neural network (CNN) [16] for guidance of rigid or affine registration. For the application of liver registration in SIRT, Alsultan et al*.* used a rigid registration method in Simplicit90Y (Mirada Medical Ltd, Oxford, UK) to register contrast-enhanced CT images to low-dose CT images for efficacy evaluation of coil embolization to acquire intrahepatic redistribution [17]. Spahr et al*.* implemented a registration framework based on normalized gradient fields for liver registration of multi-modality images and evaluated their algorithm through landmarks and deformation field analyses [18]. Nodari et al*.* used a multi-modality deformable registration algorithm in MIM SurePlan (v7.0.1; MIM software Cleveland, USA), performed by a trained medical physicist, to register the liver tumor contours from MR images to ^99m^Tc-MAA-SPECT/CT and ^90^Y-PET/CT [19]. Their study indicates that different tumor contours from anatomical and scintigraphic images have no significant impact on mean dose, and registering anatomical tumor contours to scintigraphic images is feasible for improving therapeutic strategy [19]. Besides, our in-house non-rigid liver registration regularized by a spring model was applied in our previous studies on the development of pre-treatment dosimetry [6] and evaluation of the predictive value of ^99m^Tc-MAA-based dose planning [20].

This study describes a (semi-)automatic segmentation-guided registration algorithm and evaluates its performance for registering liver contours and anatomic landmarks and its influence on dose estimation for SIRT. Our in-house image-based registration algorithm was modified for guidance by only CNN liver segmentations and by both CNN liver segmentations and manually delineated landmarks. (Semi-)automatic registration algorithms have the advantage of providing the physicians with integrated image information for more precise dosimetry, while not creating much cumbersome and time-consuming work in the clinical workflow. In our experience, the current lack of automation impedes the full use of multi-modality information. The registration can be challenging, because in the ^99m^Tc-MAA SPECT/CT protocol, the CT is acquired without contrast enhancement, and some MR images from the ^90^Y-PET/MR studies have severe artefacts. In this study, we wanted to assess (semi-)automatic liver registration and demonstrate its feasibility and value in a clinical context, so that it can contribute to a personalized and precise SIRT treatment with fewer manual interactions.

## Material and methods

### Data

The training datasets for the CNN model contain 119 CT images from Liver Tumor Segmentation (LiTS17) and SLIVER07 challenges, 30 MR T1 images from the CHAOS challenge, and 50 SIRT CT and 110 MR T1 images from our hospital.

**Fig. 1:**
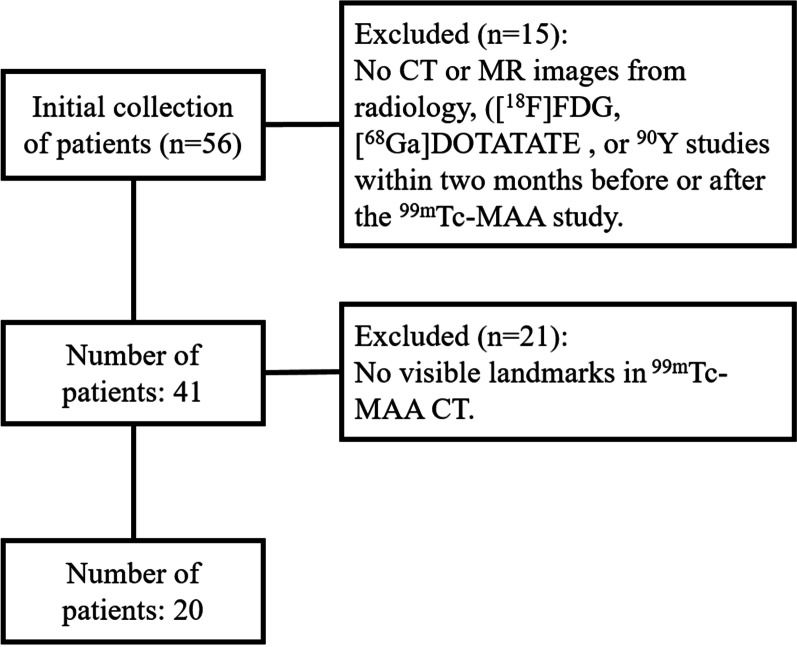
SIRT patient selection for the registration experiment

For the registration experiment, 49 CT and 26 MR images from 20 SIRT patients were selected according to several criteria. The selection procedure is presented in Fig. 1. Since the algorithm is designed for registering both CT and MR images in the pre- and post-treatment studies to the ^99m^Tc-MAA CT, there should be at least one CT and one MR from these studies in addition to the CT image from the ^99m^Tc-MAA-SPECT/CT for each patient. Since thresholding a ^99m^Tc-MAA SPECT for lesions may result in overestimation of the tumor targeting performance, landmark delineation (including lesions) for the ^99m^Tc-MAA study was performed on its CT image. ^99m^Tc-MAA CTs in our clinical routine are not contrast enhanced. Therefore, it was important to select patients with visible landmarks in ^99m^Tc-MAA CTs. After patient selection, landmarks visible in all CT and MR images for each patient were chosen and manually delineated by a nuclear medicine physician. Most landmarks were lesions, and some of them were vessel knots and metal clips. For evaluation of liver registration, liver contours were manually delineated by a trained researcher and then corrected by a radiographer with over 10 years of experience in liver delineation. For the hyper-parameter tuning of the registration algorithm, 25 CT and 14 MR images from 10 patients were randomly selected for training, 24 CT and 12 MR images from the other 10 patients were used for testing. The characteristics of the 20 SIRT patients are presented in Table 1.

**Table 1:** Characteristics of the SIRT patients for registration experiment

Characteristics	Training	Test
No. of SIRT patients	10	10
Age (y), median [range]	66.5 [46, 75]	64.5 [25, 78]
Sex (female/male)	5/5	4/6
Weight (kg), median [range]	71 [46,105]	75 [46,95]
Height (m), median [range]	1.70 [1.55, 1.74]	1.70 [1.54, 1.78]

All SIRT datasets for this research were evaluated at KU Leuven after approval by the Ethics Committee Research of UZ / KU Leuven.

### CNN structure for liver segmentation

**Fig. 2:**
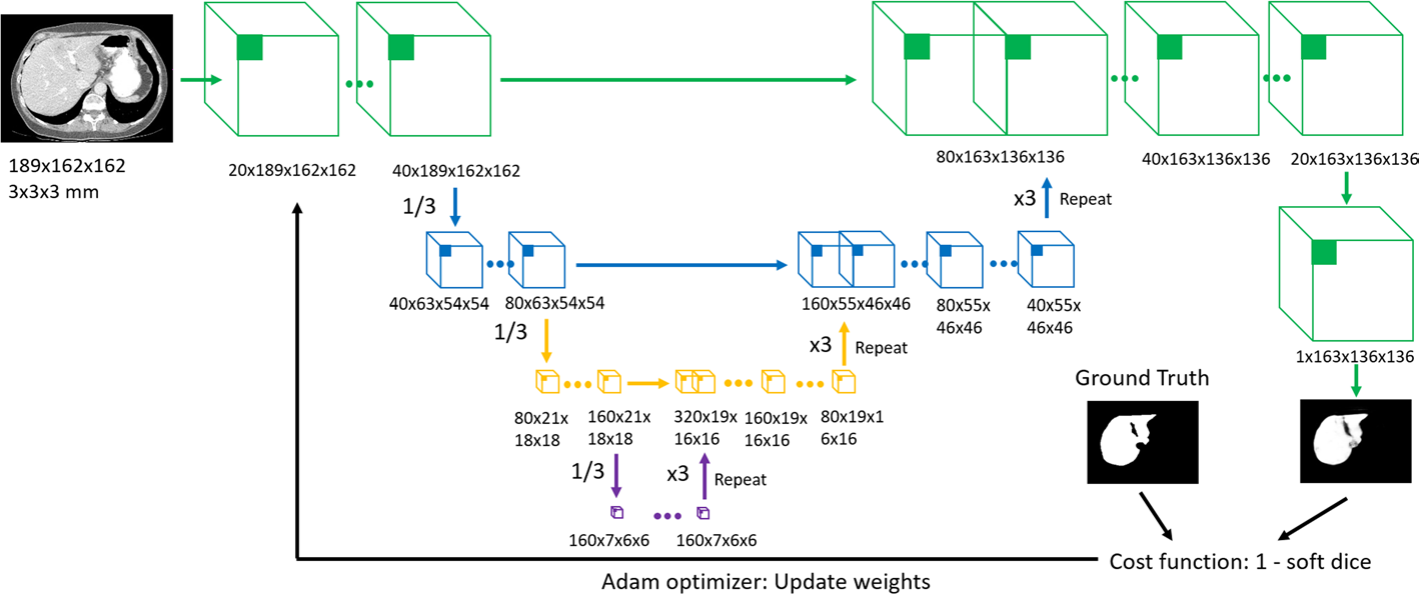
Overview of the CNN structure. The model consists of four resolution hierarchies with three skip connections. The input image has the voxel size of $3\times 3\times 3$ mm^3^. The input image size is 189 $\times 162\times 162$ , and the output image size is 163 $\times 136\times 136$ . The up- and down-sampling rate is three, and the up-sampling is implemented by repetition. The convolutional kernel size for all layers is 3 × 3 × 3

The CNN model used in the paper adopts a U-net structure [21], which was developed for biomedical image segmentation. The U-net structure (see Fig. 2), adapted for 3D images, consists of four resolution hierarchies with three skip connections to combine the high-level liver features from low-resolution hierarchies with the detailed liver features from high resolution hierarchies. Our previous research demonstrated that the CNN segmentations resulted in good segmentation quality without consuming much time and work [22].

### Segmentation-guided registration

**Fig. 3:**
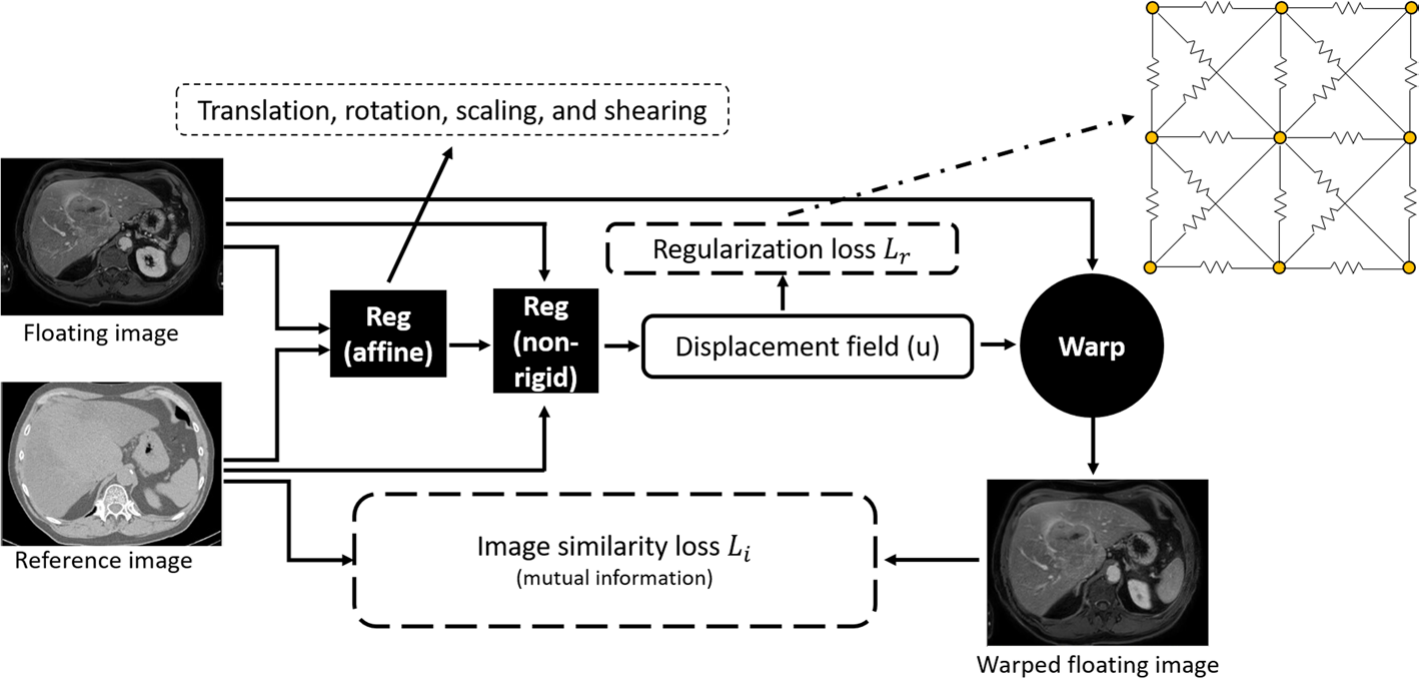
Overview of the in-house image-based registration algorithm

The in-house image-based registration algorithm (see Fig. 3) consists of affine registration followed by non-rigid registration. The image similarity metric for affine registration is computed by mutual information. The 12 affine parameters, generated after affine registration, are converted into an initial displacement field for non-rigid registration, where each element represents the displacement of the corresponding voxel in the floating image. The image similarity metric for non-rigid registration is also mutual information. To avoid topology-violating deformations during non-rigid registration, the voxel displacement is regularized through a spring model [23]. Each pair of neighboring voxels is assumed to be connected through a spring, which opposes distance changes. The spring rigidity is used to adjust the regularization power. Therefore, the image-based non-rigid registration algorithm minimizes the weighted sum of the image similarity loss (*L*_*I*_) and the regularization loss (*L*_*R*_). The computation and optimization of *L*_*I*_ and *L*_*R*_ are explained in detail in [23].

**Fig. 4:**
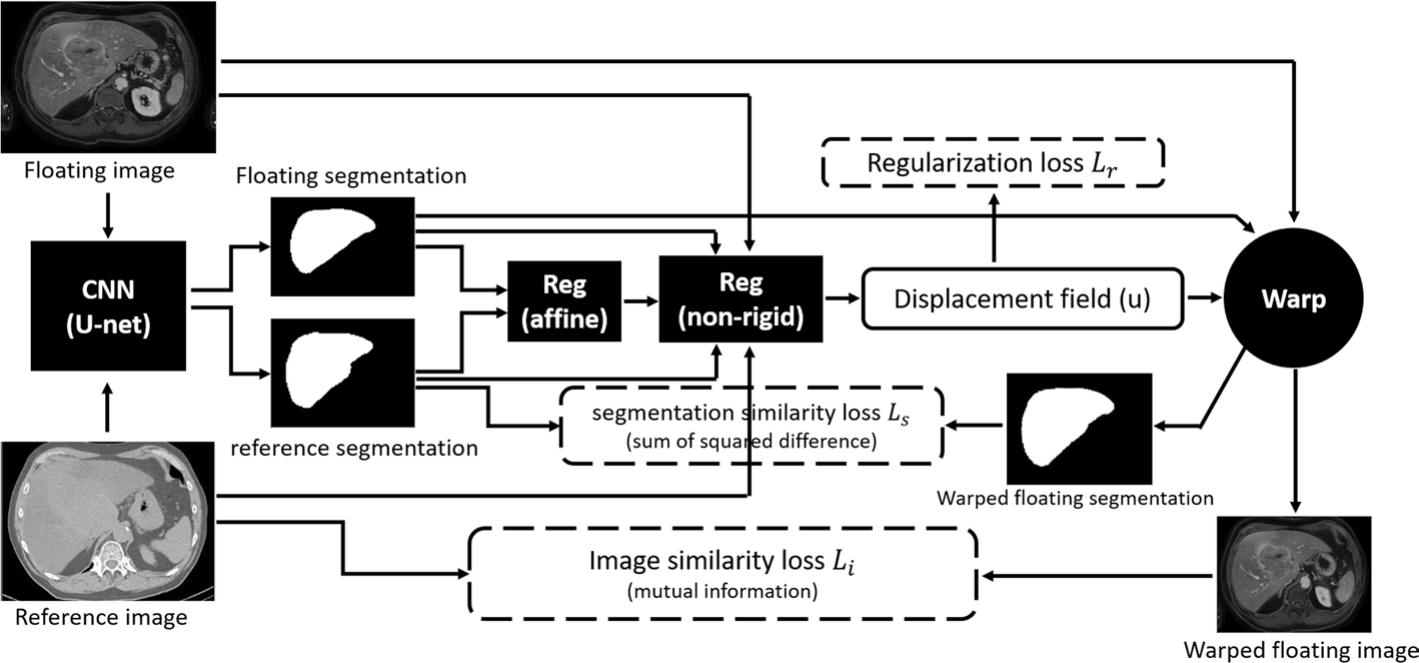
Overview of the registration algorithm guided by CNN liver segmentations

This image-based registration algorithm was extended through guidance by CNN liver segmentations (see Fig. 4). CNN liver segmentations for the reference and floating images are registered via affine transformation to provide initialization of the non-rigid registration. In the next stage, images and their CNN liver segmentations are simultaneously non-rigidly registered. The segmentation similarity loss (*L*_*S*_) is computed as the sum of squared differences between the reference and warped segmentations, representing the segmentations as binary images. The computation and optimization of *L*_*S*_ are the same as that of *L*_*I*_ when using sum of squared distance as the similarity measurement, as described in [23]. The final loss of the non-rigid registration guided by CNN liver segmentations (CNN-guided) is the weighted sum: ${w}_{I}{L}_{I}+{w}_{S}{L}_{S}+{L}_{R}$.

To explore the possibility of landmark guidance for better lesion registration, a labeled map containing both CNN liver segmentations and landmarks as two classes replaces the CNN liver segmentations in the CNN-guided algorithm with other settings unchanged. This algorithm guided by both CNN liver segmentations and landmarks (CNN&LM-guided) is compared with the image-based and CNN-guided algorithms in the following experiments.

### Experiments

#### CNN liver segmentation

The CNN model trained on CT and MR images from the public challenges, and our hospital was implemented to generate automatic liver segmentations for the 49 CT and 26 MR images from 20 SIRT patients selected for the registration experiment. The results were evaluated through the dice similarity coefficient (DSC) between the CNN liver segmentation and the ground truth liver segmentation. The DSC quantifies the overlap between the segmentations, its definition and computation are explained in [24].

#### Registration of multi-modality images

For affine registration, the results from using either images or CNN liver segmentations were compared. For non-rigid registration, 1D (${w}_{S}=0$) and 2D grid searches were implemented to find the optimal weights without and with guidance of CNN liver segmentations. The non-rigid registration was initialized by either image-based or CNN-based affine registration. To evaluate the value of landmark guidance for better lesion registration, 2D grid searches were implemented to find the optimal weights for the CNN&LM-guided non-rigid registration initialized by the CNN&LM-based affine registration. Each liver registration was evaluated by the root mean squared distance (RMSD) of mean surface distance (MSD) between liver contours and mass center distance (MCD) between landmarks: $\mathrm{RMSD}=\sqrt{({\mathrm{MSD}}^{2}+{\sum }_{i=1}^{{N}_{\mathrm{landmarks}}}{\mathrm{MCD}}_{i}^{2})/(1+{N}_{\mathrm{landmarks}})}$. Mean surface distance is designed to measure the contour difference between two segmentations [25]. Since a landmark can appear with different shapes and volumes in images of different modality, mass center distance, which is independent of shape and volume changes, is used to evaluate landmark registration. The root mean square of RMSDs (RRMSD) from all liver registrations was used to evaluate each pair of weights. The optimal weights, with the lowest RRMSD, were found through grid searches on the training datasets for CT to CT and MR to CT registrations, respectively. After that, different registration settings were compared on the test datasets through RMSD and RRMSD.

#### Dose estimation

The CNN- and CNN&LM-guided registrations using the optimal weights were implemented to generate registered landmarks (including lesions) for the floating CT and MR images (registered floating landmarks) from the test datasets for comparison with the landmarks delineated on ^99m^Tc-MAA CTs (reference landmarks). A five-scale Likert score, with its criteria presented in Table 2, was used for grading the floating landmarks registered to the reference landmarks. Dose estimation using the registered floating lesions from the CNN- and CNN&LM-guided algorithms was compared with dose estimation using the reference lesions through the mean dose and the volume percentage receiving at least 70 (V70) and 100 Gy (V100) in the lesion. The absorbed doses of 70 and 100 Gy illustrate an intermediate and high tumor response probability. The injected activities prescribed for the left and right LPTs for each patient were used for dose estimation. LPTs are delineated on CBCTs in our clinical workflow. Dose estimation computed on the left and right LPTs requires registration of CBCT to ^99m^Tc-MAA-SPECT/CT, which introduces extra potential sources of registration errors. To focus on evaluating the influence of CT and MR registration on dose estimation in this study, the injected activities for the left and right LPTs are summed and distributed in a fractional uptake map generated by normalizing all counts of the ^99m^Tc-MAA SPECT within the manually delineated liver contour. The computation of the absorbed dose was based on the local deposition model.

**Table 2:** Likert score criteria for scoring landmark registration

Score	Criteria
1	Major misalignment exists for the registration. Major impact on dosimetry is expected. Dosimetry results are deemed unreliable
2	Pronounced misalignment exists for the registration. Substantial impact on dosimetry is expected
3	Moderate misalignment exits for the registration. Moderate impact on dosimetry is expected
4	Little misalignment exists for the registration. No significant impact on dosimetry is expected
5	Near perfect alignment for the registration. No intervention is warranted and dosimetry is deemed reliable

## Results

### CNN liver segmentation

**Fig. 5:**
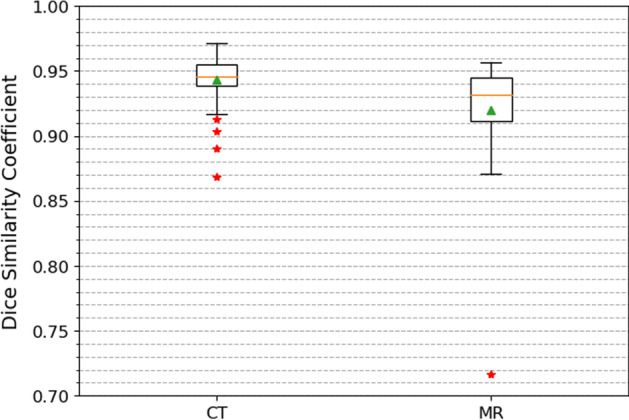
DSCs between manual liver segmentations and CNN liver segmentations of 49 CT and 26 MR images from the 20 SIRT patients selected for the registration experiment. The orange line, green triangle, and red stars represent median, mean, and outliers, respectively. The box corresponds to the first and third quartiles, and the whiskers give the range (except for the outliers)

The DSCs between CNN liver segmentations of CT and MR images from 20 SIRT patients for registration and manual liver segmentations are presented in Fig. 5. The median DSCs for CT and MR images are around 0.95 and 0.93, respectively. The DSCs for MR images have an outlier with a very low value of 0.72. Through visual inspection, the CNN segmentation for this MR image misses a large part of the liver volume and is incapable of registration guidance. Therefore, the case involving this MR image was excluded for MR to CT registration.

### Registration of multi-modality images

**Fig. 6:**
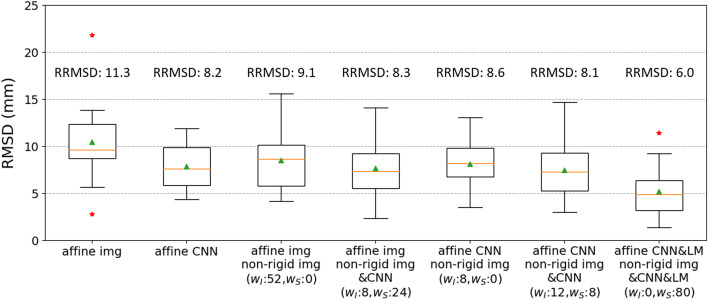
RMSDs and RRMSDs of the test datasets (14 floating CT images) for comparison of image-based, CNN-guided, and CNN&LM-guided CT to CT registrations

The results of CT to CT registrations are presented in Fig. 6, using the optimal weights for each deformable registration. The RRMSD for CNN-based affine registration is 3.1 mm (27%) smaller than that for image-based affine registration. The image-based non-rigid registration increases the RRMSD for CNN-based affine registration by 0.4 mm (5%). The RRMSD for CNN-guided affine and non-rigid registration^1^ is 1.0 mm (11%) smaller than that for image-based affine and non-rigid registration. The CNN&LM-guided affine and non-rigid registration^2^ has a RRMSD 2.1 mm (26%) smaller than the CNN-guided one.

**Fig. 7:**
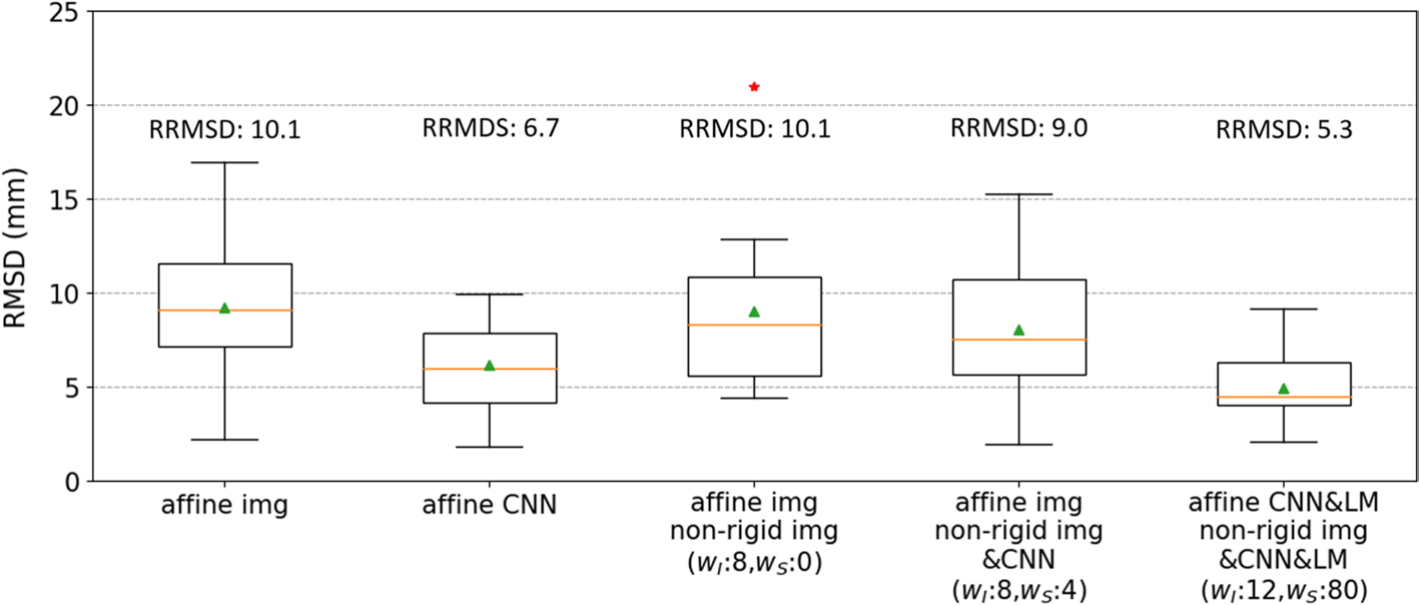
RMSDs and RRMSDs of the test datasets (12 floating MR images) for comparison of image-based, CNN-guided, and CNN&LM-guided MR to CT registrations

The results of MR to CT registrations are presented in Fig. 7, using the optimal weights for each deformable registration. The CNN-based affine registration has a RRMSD 3.4 mm (34%) smaller than the image-based one does. The optimal *w*_*I*_ and *w*_*S*_ for CNN-guided non-rigid registration initialized by CNN-based affine registration were both zero. Therefore, the optimal performance of CNN-guided affine and non-rigid registration is the same as that of the CNN-based affine registration. This indicates that both image-based and CNN-guided non-rigid registration degrade the results of CNN-based affine registration. The RRMSD for CNN&LM-guided affine and non-rigid registration is 1.4 mm (21%) smaller than that for CNN-based affine registration.

### Dose estimation

According to the results in the registration experiment, the optimal performance for CNN&LM- and CNN-guided CT to CT registration and CNN&LM-guided MR to CT registration were achieved when using both affine and non-rigid registrations, while the optimal performance for CNN-guided MR to CT registration was achieved when using only affine transformation. These settings using the optimal weights were used to generate the registered landmarks for dose estimation.

#### Registration of CT to ^99m^Tc-MAA CT

**Fig. 8:**
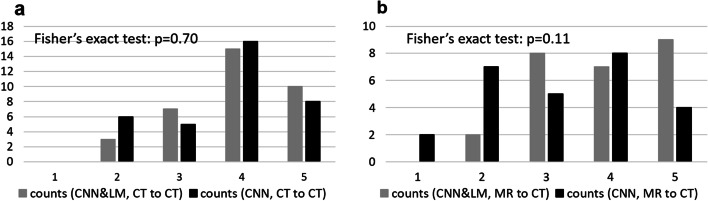
Likert scores for the registered landmarks of floating CT (**a**) and MR (**b**) images using the CNN&LM-guided and CNN-guided registrations

**Fig. 9:**
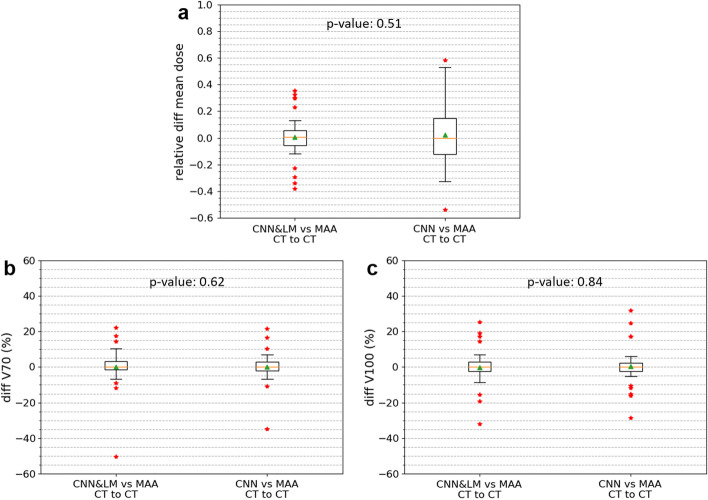
Relative difference of mean dose (**a**) and difference of V70 (**b**) and V100 (**c**) between the reference and registered floating lesions using the CNN&LM-guided or CNN-guided CT to CT registration. MAA: the reference lesions. CNN&LM: the floating lesions registered by the CNN&LM-guided registration. CNN: the floating lesions registered by the CNN-guided registration. The relative difference of mean dose is computed by (mean dose (floating) – mean dose (reference)/mean dose (reference)). The difference of V70 and V100 is computed by (V70 or V100 (floating) – V70 or V100 (reference))

**Fig. 10:**
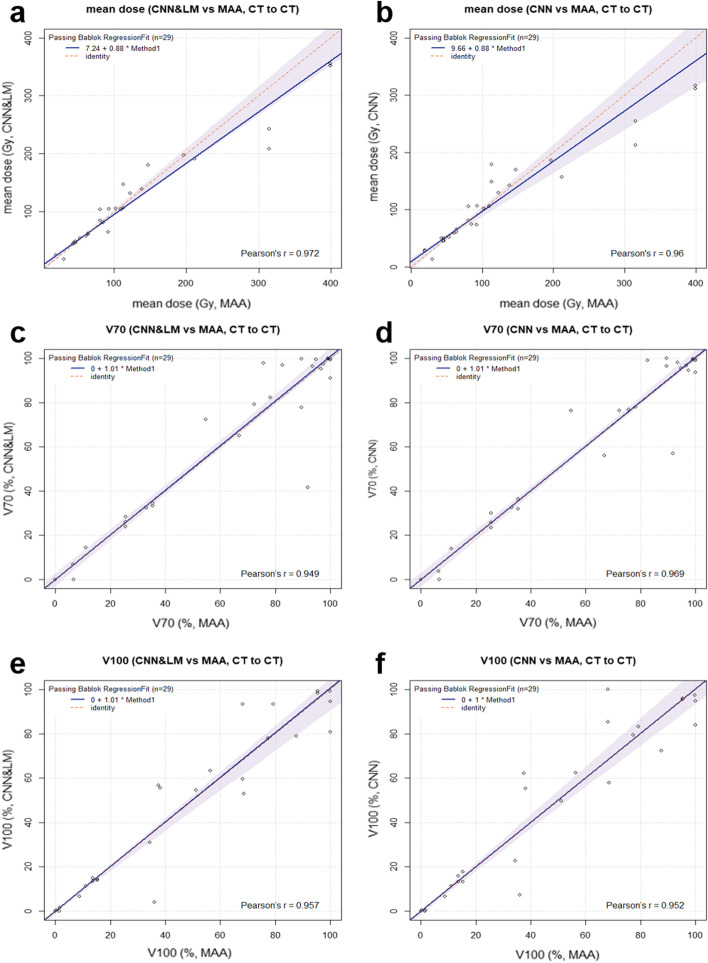
Passing-Bablok plots for the mean dose, V70, and V100 estimated on the floating CT lesions registered by the CNN&LM- or CNN-guided algorithm versus the mean dose, V70, and V100 estimated on the reference lesions. MAA: the reference lesions. CNN&LM: the floating lesions registered by the CNN&LM-guided registration. CNN: the floating lesions registered by the CNN-guided registration

The Likert scores for registration of all landmarks (lesions, vessels, clips, etc.) are presented in Fig. 8a. Over two thirds of registered landmarks had a score of at least 4, which means little or almost no misalignment and insignificant impact on dosimetry. There were in total 29 lesions from 10 SIRT patients used for dose estimation. The relative difference of mean dose and the difference of V70 and V100 between the reference and registered floating lesions using the CNN&LM- and CNN-guided registrations are shown in Fig. 9. There were 59% and 45% of lesions having an absolute relative difference of mean dose smaller than 10% for the CNN&LM- and CNN-guided registrations, respectively. Around 79% (76%) and 83% (69%) of lesions have an absolute V70 (V100) difference smaller than 10% for the CNN&LM- and CNN-guided registrations, respectively. As shown in Fig. 10, the mean dose, V70, and V100 for the floating lesions registered by both the CNN&LM- and CNN-guided algorithms had a strong correlation ($r\ge 0.95$) with the mean dose, V70, and V100 for the reference lesions.

#### Registration of MR to ^99m^Tc-MAA CT

**Fig. 11:**
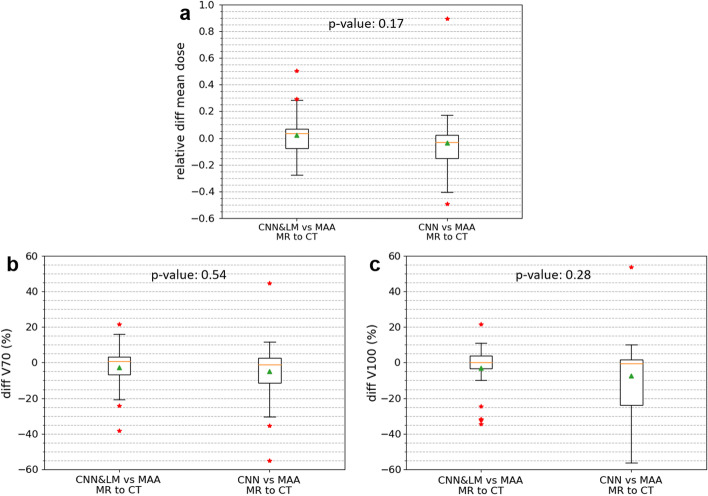
Relative difference of mean dose (**a**) and difference of V70 (**b**) and V100 (**c**) between the reference and registered floating lesions using the CNN&LM-guided or CNN-guided MR to CT registration. MAA: the reference lesions. CNN&LM: the floating lesions registered by the CNN&LM-guided registration. CNN: the floating lesions registered by the CNN-guided registration. The relative difference of mean dose is computed by (mean dose (floating) – mean dose (reference)/mean dose (reference)). The difference of V70 and V100 is computed by (V70 or V100 (floating) – V70 or V100 (reference))

**Fig. 12:**
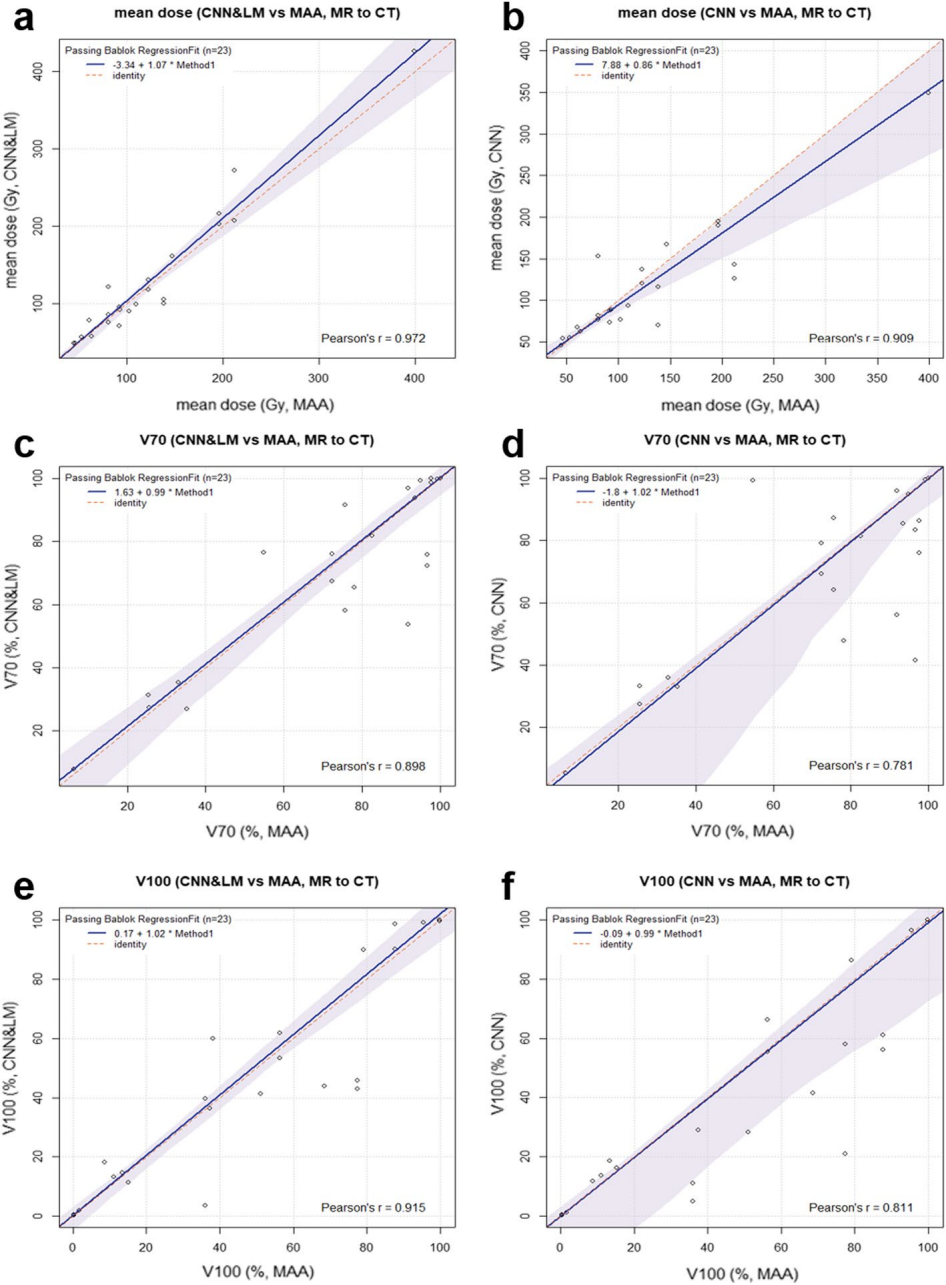
Passing-Bablok plots for the mean dose, V70, and V100 estimated on the floating MR lesions registered by the CNN&LM- or CNN-guided algorithm versus the mean dose, V70, and V100 estimated on the reference lesions. MAA: the reference lesions. CNN&LM: the floating lesions registered by the CNN&LM-guided registration. CNN: the floating lesions registered by the CNN-guided registration

According to the Likert scores for all registered landmarks shown in Fig. 8b, around 92% and 63% of lesions registered by the CNN&LM- and CNN-guided algorithms had a score equal to or better than “moderate misalignment and impact on dosimetry”, respectively. There were in total 23 lesions from 10 SIRT patients for dose estimation. The relative difference of mean dose and the difference of V70 and V100 between the reference and registered floating lesions are presented in Fig. 11. Around 70% and 43% of lesions had an absolute difference of mean dose smaller than 10% for the CNN&LM- and CNN-guided registrations, respectively. The CNN&LM- and CNN-guided registrations have around 70% (70%) and 61% (61%) of lesions with an absolute V70 (V100) difference smaller than 10%, respectively. The correlation of mean dose, V70, and V100 between the reference and registered floating lesions is presented in Fig. 12. A weaker correlation ($r<0.90$) of V70 and V100 for the CNN-guided registration is observed than the correlation of mean dose for the CNN&LM- and CNN-guided registrations and of V70 and V100 for the CNN&LM-guided registration.

**Fig. 13:**
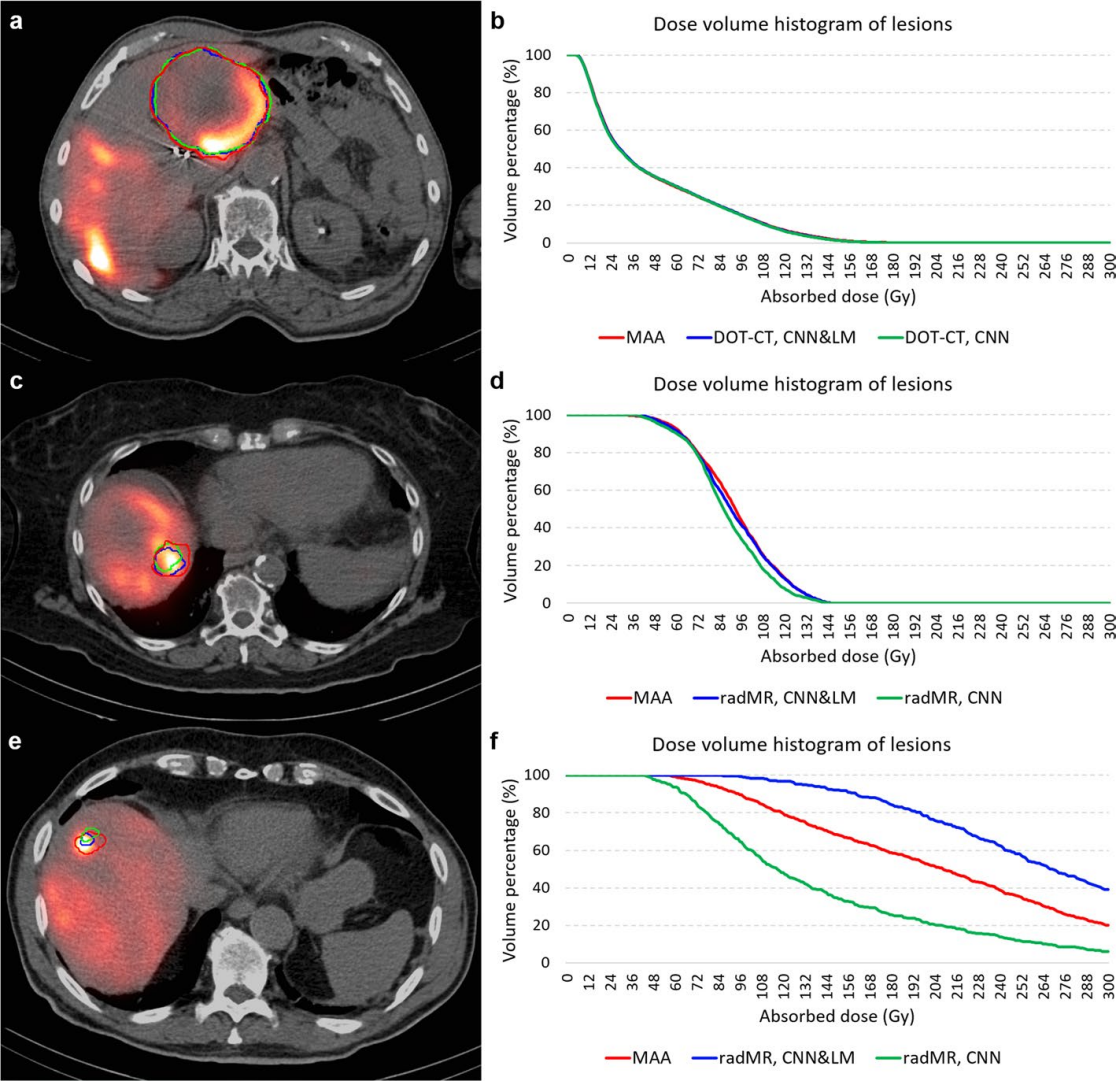
Examples of the reference and floating lesions registered by the CNN&LM- and CNN-guided methods and their dose volume histograms from three patients. The red, blue, and green contours represent the reference lesion (MAA), the floating lesion registered by the CNN&LM-guided method (CNN&LM), and the floating lesion registered by the CNN-guided method (CNN). **a**, **b** the registered floating lesions from the CT of the DOTATATE (DOT) study; CNN&LM: score is 5, relative difference of mean dose is − 0.1%, the differences of V70 and V100 are 0.3% and − 0.1%, CNN: score is 5, relative difference of mean dose is − 0.9%, the differences of V70 and V100 are 0.2% and − 0.4%. **c**, **d** the registered floating lesions from the radiology MR (radMR); CNN&LM: score is 3, relative difference of mean dose is − 1.3%, the differences of V70 and V100 are − 0.7% and − 1.1%, CNN: score is 2, relative difference of mean dose is − 5.2%, the differences of V70 and V100 are − 1.0% and − 8.4%. **e, f** the registered floating lesions from radMR; CNN&LM: score is 3, relative difference of mean dose is 28.4%, the differences of V70 and V100 are 2.5% and 11.1%, CNN: score is 2, relative difference of mean dose is − 32.5%, the differences of V70 and V100 are − 11.1% and − 26.5%

Some examples of the reference and floating lesions registered by the CNN&LM- and CNN-guided methods and their dose volume histograms (DVHs) are presented in Fig. 13.

## Discussion

### Registration of multimodality images

The CNN-based affine registration of CT to CT and MR to CT improves the RRMSD by 3.1 mm (27%) and 3.4 mm (34%), respectively, compared with the image-based affine registration. This substantial decrease shows the advantage of using CNN liver segmentations for affine registration without introducing non-affine deformation. The RRSMD increases by 0.4 mm (5%) when the CNN-guided affine registration is followed by the image-based non-rigid registration for CT images. The CNN-guided non-rigid registration of CT images decreases the RRMSD for the CNN-based affine registration by 0.1 mm (1%). This indicates the slightly negative influence of using only images (including non-contrast-enhanced ^99m^Tc-MAA CT) and the limited improvement of using CNN liver segmentations for non-rigid registration of CT images, given the good initialization provided by the CNN-based affine registration. The optimal weights found for the CNN-guided affine and non-rigid MR to CT registration were both zero indicating that using both images and CNN liver segmentations for non-rigid MR to CT registration could not improve the results from the CNN-based affine registration. This might be caused by relatively poorer CNN liver segmentations for MR images than for CT images. Some CNN liver segmentations for MR images were found to miss some low-intensity lesion regions. This can cause big misalignment in these regions due to liver surface matching guided by CNN liver segmentations during the non-rigid registration. Nevertheless, the CNN-guided registration improves the RRMSD by 1.0 mm (11%) and 3.4 mm (34%) for the floating CT and MR images compared with the image-based registration. Even if there might be errors in CNN liver segmentations compared to the “ground truth”, these unedited CNN segmentations are still helpful for improving the image-based registration. This enables the automation of the liver-segmentation-guided registration without the need of extra manual correction for CNN liver segmentations.

Through landmark guidance, the RRMSD is decreased by 2.1 mm (26%) and 1.4 mm (21%) compared with the CNN-guided registration for the floating CT and MR images, respectively. Since we currently don’t have an automatic lesion segmentation tool for CT and MR images, manually delineated landmarks were used for both registration guidance and evaluation. This might cause a self-fulfilling effect for registration evaluation. However, it does not change the feasibility of using landmarks for better registration, since manually delineated landmarks can be taken as perfect automatic lesion segmentations. This indicates that developing automatic lesion segmentation would be beneficial for registration guidance. Besides, manually delineated lesions approved by the physician are usable in the clinical context.

### Dose estimation

A strong correlation ($r>0.9$) of mean dose estimation existed between the reference and floating lesions registered by the CNN&LM- and CNN-guided registrations. Landmark guidance for the CNN-guided registration resulted in a smaller difference of mean dose for CT to CT registration than for MR to CT registration. Since mean dose is computed on the volume level, it appears less sensitive to contour changes than the voxel-level dosimetry.

For the voxel-level dosimetry, the CNN&LM- and CNN-guided registrations had around 79% (76%) and 83% (69%) of lesions with an absolute V70 (V100) difference between the reference and floating CT lesions smaller than 10%, respectively. A very strong correlation ($r\ge 0.95$) of V70 and V100 existed between the reference and floating CT lesions for the two methods. Landmark guidance for CT to CT registration made small improvement for the voxel-level dosimetry, which was also reflected in the Likert scores given by the physician. Around 70% (70%) and 61% (61%) of MR lesions had an absolute V70 (V100) difference smaller than 10% for the CNN&LM- and CNN-guided registrations, respectively. A weaker correlation ($r<0.82$) of V70 and V100 was observed between the reference lesions and the floating MR lesions for the CNN-guided registration than for the CNN&LM-guided registration. Landmark guidance for MR to CT registration helped decrease the discrepancy of the voxel-level dose estimation caused by lesion registration.

It was found that the relative difference of mean dose and the difference of V70 and V100 were smaller than 10% for most lesions with a volume over 50 cc. It is reasonable that small lesions with a small shift can create a relatively large voxel change. Besides, lesions delineated on images of different modality can appear with diverse shape and volume, due to different lesion information expressed in different images or tumor development. Small volumes and large shape and volume differences accounted for a V70 (V100) difference over 10% for 4 (3) out of 6 (7) and 3 (4) out of 5 (9) floating CT lesions registered by the CNN&LM- and CNN-guided algorithms and for 4 (2) out of 7 (7) and 5 (3) out of 9 (9) floating MR lesions registered by the CNN&LM- and CNN-guided algorithms. Good lesion registration does not ensure small difference of dose estimation, since the small size and large shape and volume difference are the other two critical factors with significant impact on dose estimation. It is difficult to eliminate the shape and volume difference between lesions delineated on different images, since each modality reflects a different aspect of lesion appearance. Therefore, it is beneficial to co-register all multi-modality images for joint lesion delineation by the physician, to approach the ground truth delineation by making full use of all information.

Poor lesion registration does not necessarily lead to significant changes of dose estimation. As presented in Fig. 13c, d, the lesion (green) of radiology MR registered by the CNN-guided method, scored with 2, does not have a good overlap with the reference lesion (red), while both lesion contours include most of the high-uptake region. The relative mean dose difference and the V70 and V100 difference between the reference and registered lesions are − 5.2%, − 1.0%, and − 8.4%, respectively. As long as the reference and registered lesions include a similar area of high- and low-uptake regions, the difference of dose estimation can be insignificant despite of poor registration. This indicates that the volume percentage does not necessarily reflect the true energy deposition for each voxel.

In our standard workflow, the liver and lesions are manually delineated on the anatomic image by using the delineation tools from a clinical software package used by the physician for SIRT planning. After that, the delineated volumes of interest (VOIs) are mapped to the ^99m^Tc-MAA SPECT/CT by using the manual or semi-automatic registration tools of the software to register the anatomic image to the ^99m^Tc-MAA CT. To shorten the processing time, the physician delineates the VOIs directly on the ^99m^Tc-MAA SPECT in selected cases, obviating the need for registration. However, proper registration of anatomical images to the ^99m^Tc-MAA SPECT/CT allows lesion delineation on the anatomical images and correlation to the SPECT findings, as recommended in recent international guidelines for SIRT [26]. The standard workflow requires the physician’s interaction during the entire process. In general, the time to complete the standard workflow is around 30 to 45 min. The segmentation-guided workflow consists of liver segmentation, registration, and lesion delineation. To facilitate clinical application and evaluation of these new tools, we have incorporated the entire workflow into the clinical software platform. Liver segmentation is fully automated by the CNN. It takes no more than 5 min for the trained CNN model to generate one liver segmentation by a CPU-based computation server. After that, the CNN liver segmentations are checked and corrected, if necessary, by the physician to ensure its usability for registration guidance, which takes 1 to 5 min. The segmentation-guided registration algorithm is performed by a CPU-based server without parallel computation, which takes around 15 min for each registration in general. Since the registration workflow is fully automated without manual interaction needed, the processing time is acceptable for routine clinical use. It could be speeded up by implementing parallel computation. Lesion delineation is manually performed by using the delineation tools of the clinical software, which takes around 10 min and needs to be automated in the future. In total, the segmentation-guided workflow can take around 30 min. The automated processing takes 20 min, which does not need the physician’s interaction. This makes the segmentation-guided workflow a useful tool for the physician.

In summary, the performance of the CNN&LM- and CNN-guided registrations makes them useful tools for SIRT treatment planning and verification. The deployment of these semi- and automatic registration tools would allow for dose prediction and measurement based on multi-modality images without introducing much manual interaction and workload, which currently impede the application of image analysis tools in the clinical workflow. The pre- and post-treatment studies contain many images with relatively poor quality, including non-contrast-enhanced CTs from the ^99m^Tc-MAA study and MR with severe shading or bias artifacts from the ^90^Y-PET/MR. Nevertheless, these registration algorithms can produce reasonably good results for these low-quality images giving them practical value for clinical application. Based on these results, we will study the development of an automatic liver lesion segmentation method for fully automatizing the CNN&LM-guided registration. The clinical influence of these registration methods remains to be fully evaluated in a daily SIRT workflow from a volume-level and voxel-level perspective.

## Conclusion

Registration guidance using CNN liver segmentations and landmarks greatly improved the performance of the in-house image-based registration. The CNN&LM- and CNN-guided registrations for CT and MR images can be used for the volume-level dosimetry, since the mean doses obtained from the reference and floating lesion contours were very similar. A small V70 and V100 change for most lesions of the floating CT images using the CNN&LM- and CNN-guided registrations demonstrates the feasibility of their application to the voxel-level dosimetry with the physician’s checkup. Landmark guidance is needed for the CNN-guided MR to CT registration to be applied to the voxel-level dosimetry. As a result, the CNN&LM- and CNN-guided registration algorithms could become valuable semi- and automatic tools applicable for SIRT dosimetry based on integration of multi-modality information.

## Data Availability

Due to GDPR, we cannot make the data publicly available.

## Footnotes

1 “The CNN-guided affine and non-rigid registration” always refers to the CNN-based affine and CNN-guided non-rigid registration.

2 “The CNN&LM-guided affine and non-rigid registration” always refers to the CNN&LM-based affine and CNN&LM-guided non-rigid registration.

## Code availability

The code used in this study may be made available upon request from the corresponding author.
